# Transcriptome Analysis Reveals a Signature Profile for Tick-Borne Flavivirus Persistence in HEK 293T Cells

**DOI:** 10.1128/mBio.00314-16

**Published:** 2016-05-24

**Authors:** Luwanika Mlera, Jennifer Lam, Danielle K. Offerdahl, Craig Martens, Daniel Sturdevant, Charles V. Turner, Stephen F. Porcella, Marshall E. Bloom

**Affiliations:** a Laboratory of Virology, Rocky Mountain Laboratories, National Institutes of Allergy and Infectious Diseases, National Institutes of Health, Hamilton, Montana, USA; b Genomics Unit, Research Technologies Section, Rocky Mountain Laboratories, National Institute of Allergy and Infectious Diseases, Hamilton, Montana, USA

## Abstract

Tick-borne flaviviruses (TBFVs) cause febrile illnesses, which may progress to severe encephalitis and/or death in humans globally. Most people who recover from severe acute disease suffer from debilitating neurological sequelae, which may be due to viral persistence, infection-induced neurological cell damage, host response, or some combination of these. Acute TBFV infection of human embryonic kidney (HEK) 293T cells *in vitro* results in the death of >95% of infected cells by day 5. However, replacing cell growth medium allows surviving cells to repopulate and become persistently infected for extended periods of time. The mechanisms responsible for initiation and maintenance of viral persistence remain vague. We subjected the HEK 293T cell transcriptome to deep sequencing to identify genes differentially expressed during acute infection and persistent infection. A total of 451 genes showed unique significant differential expression levels in persistently infected cells relative to the acute phase of infection. Ingenuity Pathway Analysis results suggested that the expression of prosurvival oncogenes *AKT2* and *ERBB2* was upregulated in persistently infected cells, whereas proapoptotic genes, such as *Bad* and the beta interferon 1 (IFN-β1) gene, were downregulated. Genes encoding antiviral cytokines such as the CCL5, tumor necrosis factor alpha (TNF-α), and CXCL10 genes were upregulated during the acute phase, but the same genes were relatively quiescent in persistently infected cells. Exogenous induction of apoptosis demonstrated that persistently infected cells were resistant to apoptosis in a dose-dependent manner. In summary, the differential transcriptome profiles of acute-phase compared to persistently infected HEK 293T cells demonstrated an evasion of apoptosis, which may be critical for a chronic TBFV infection state. These results provide a basis for further study of the mechanisms of TBFV persistence.

## IMPORTANCE

Tick-borne flaviviruses (TBFVs) cause life-threatening encephalitic disease in humans worldwide. Some people who recover from severe disease may suffer prolonged neurological symptoms due to either virus- or host response-induced cell damage or a combination of the two that are linked to viral persistence. By examining the genes that are significantly differentially expressed in acute TBFV infection versus persistent TBFV infection, we may be able to find the molecular basis of viral persistence. Here we used deep sequencing of the host cell transcriptome to discover that the expression levels of prosurvival genes were upregulated in persistently infected cells relative to acute TBFV infections whereas the expression levels of genes that promote programmed cell death were downregulated. In addition, persistently infected cells were also resistant to exogenous chemical induction of cell death, in a dose-dependent manner, compared to uninfected cells. Our results pave the way for further studies aimed at understanding the precise mechanisms of TBFV persistence.

## INTRODUCTION

Vector-borne flaviviruses (VBFVs) are distributed worldwide and have a significant impact on human morbidity and mortality (1–3). VBFVs are classified into tick-borne flaviviruses (TBFVs) and mosquito-borne flaviviruses (MBFVs). Members of the TBFV group include Powassan virus (POWV) and its close relative, deer tick virus (DTV), tick-borne encephalitis virus (TBEV), and Omsk hemorrhagic fever virus (OHFV). The MBFVs include dengue virus (DENV), West Nile virus (WNV), Japanese encephalitis virus (JEV), and yellow fever virus (YFV). VBFVs are considered emerging and reemerging viruses as evidenced by the dramatic 400% increase in TBFV disease observed in the 1973–2001 period in Europe, as well by as the appearance of VBFVs in new geographic areas, such as the epidemic appearance of WNV in the United States in 1999 and Zika virus in regions outside Africa and Asia, and the identification of new viruses, such as Alkhurma hemorrhagic fever virus (4–9). Many of the VBFV agents must be studied under biosafety level 3 (BSL-3) or BSL-4 conditions, but the naturally attenuated Langat virus (LGTV) is a convenient model for studying TBFV biology at BSL-2.

Despite the wide diversity in VBFVs, the genome organizations and virion structures are quite similar. An 11-kb positive single-stranded RNA [ss(+)RNA] genome is flanked by 5′ and 3′ noncoding regions and codes for a single large polyprotein (10, 11). Viral and host proteases cleave the polyprotein into 3 structural proteins (C, prM/M, and E) and 7 nonstructural proteins (NS1, NS2A, NS2B, NS3, NS4A, NS4B, and NS5) (10–13). The 3 structural proteins form a mature icosahedral enveloped virion with a diameter of ~500 Å, the entire surface of which is comprised of the E protein (10, 11, 14). Several of the nonstructural proteins are known to play critical roles in replication and evasion of the host cell innate immune response (15, 16).

TBFV infection in humans is usually acquired through a tick bite, but alimentary infection following consumption of unpasteurized milk is also documented (17–19). Infection generally leads to a self-limiting acute febrile illness but may progress into a secondary phase characterized by invasion of the central nervous system. Consequently, clinical signs of the second phase of disease are neurological, with encephalitis and meningitis being the most common (20). Although it is well known that flavivirus replication broadly causes inflammation, cell lysis, and dysfunction, the mechanism(s) by which the febrile phase transforms into life-threatening neurological disease remains poorly characterized (2, 3, 17). Furthermore, 30% to 60% of individuals who survive the encephalitic stage of TBFV infection develop prolonged debilitating neurological symptoms, which may be the result of direct viral cytopathology during acute infection, persistent viral infection, the host anti-viral response, or a combination of these (2, 3, 16, 21–23).

One aspect of TBFV biology that has received little attention as a determinant of disease is viral persistence (21). Due to our interest in defining the viral and host factors that enable these highly lytic viruses to initiate and maintain a noncytopathic persistent infection, we developed an *in vitro* model for TBFV persistence (24). LGTV infection of human embryonic kidney (HEK) 293T cells *in vitro* leads to an acute lytic crisis, with massive cell death seen by day 5. However, a small fraction (<5%) of the infected cells survive and remain persistently infected beyond 30 weeks (24). We determined by single-cell isolation that this surviving fraction of cells is not a result of altered expression to begin with (L. Mlera, unpublished results). The persistently infected cultures do not undergo subsequent lytic crises and morphologically resemble uninfected cells. When we performed detailed examination of the virus genome by segmental PCR and next-generation sequencing (NGS), it was evident that there were no changes in the viral RNA sequence during acute infection or at the time that persistence was initiated. However, a unique population of defective interfering virus particles (DIPs) was associated with maintenance of the persistent infection (24). Thus, although DIPs are a feature of persistent infection in cell culture, they are not required to initiate a persistent infection.

One interpretation of these results is that a small population of infected cells was able to evade the cytopathic lytic crisis and support a persistent infection. In order to examine this hypothesis, we employed transcriptome analysis using NGS technology to compare gene expression patterns in cell cultures undergoing virus-induced lysis with the patterns in those that become persistently infected. Our results suggested that downregulation of the expression of proapoptotic genes occurs and is maintained over time. Suppression of proapoptotic genes and pathways may play a role in the establishment of TBFV persistence in mammalian cells *in vitro*.

## RESULTS

### Early transcriptome profile changes in HEK 293T cells exposed to heat-inactivated Langat virus.

The overriding aim of these studies was to elucidate transcriptome profile changes in LGTV-infected cells, comparing results of acute and persistent infections. However, we first wanted to distinguish whether early transcriptional events were ascribable to the inbound virus particle or were rather a consequence of early infection. Therefore, we compared the transcriptome profiles of cells exposed to inactivated LGTV for 12 h with those of cells exposed to infectious LGTV for the same length of time, both relative to the profile determined for mock-infected cells.

Compared to mock-infected cells, inactivated LGTV significantly (>2-fold change; false-discovery rate [*q*] of <0.05) affected the differential expression levels of 2,239 genes, whereas infectious LGTV perturbed the expression of 2,540 genes. Scrutiny of the data showed that all of the 2,239 genes that were perturbed by inactivated virus were also affected by infectious LGTV. Thus, components of the inbound virus particle are responsible for early changes in the transcription of a number of genes. However, differences in the degree of fold change in up- or downregulation were noted; i.e., a greater fold change in gene expression levels was induced by infectious virus. A total of 74% of the 2,239 genes were upregulated, whereas 26% of the genes were downregulated in expression. In both cases, the genes showing increased expression relative to mock-infected cells included genes related to innate immune response and gamma interferon (IFN-γ) signaling, such as *IFIT1* (encoding interferon-induced protein with tetracopeptide repeat 1; 2.7-fold change), the interleukin-12A (*IL12A*) gene (2.3-fold change), and *IL23A* (2.7-fold change). In contrast, genes showing decreased expression levels relative to those seen with mock-transfected cells included the mitogen-activated protein kinase kinase kinase (*MAP3K9*) gene (−2.2-fold change) and *CXCL1* (chemokine [C-X-C motif] ligand 1) (−9.5-fold change), which plays a role in inflammation. Interestingly, the 301 genes that were uniquely altered in cells exposed to infectious virus included the gene encoding NF-κB inhibitor alpha (*NF-ĸBIA*; −2.5-fold change), which is involved in innate immune responses to viral infection.

We evaluated these data using Ingenuity Pathway Analysis (IPA; QIAGEN, Redwood, CA). This Web-based functional analysis tool for identifying signaling pathways can predict downstream effects on biological processes as well as activation or inhibition of transcription factors from high-throughput gene expression data (25). The canonical pathways most significantly (ranked by *z* score) associated with both infectious and heat-inactivated LGTV at 12 h postinfection (hpi) were those corresponding to EIF2 signaling, oxidative phosphorylation, mitochondrial dysfunction, regulation of EIF4 and p70s6k signaling, and mTOR signaling. More extensive IPA indicated that pathways that were enriched for these genes mainly involved functions associated with viral infection, cell proliferation, and cell polarization.

### Global transcriptome profiles associated with acute Langat virus infection.

We next characterized the differential gene expression levels associated with the temporal progression of acute LGTV infection by comparing the transcriptome profiles of LGTV-infected cells at several time points relative to those determined at 12 h postinfection (hpi). The comparison indicated that the following numbers of genes showed significant differential expression levels (>2-fold change; *q* < 0.05); 1,474, 1,377, 3,085, and 5,033 genes at 24, 48, 72, and 96 hpi, respectively. Thus, there was a general increase in the number of differentially expressed genes as the acute infection proceeded from 12 hpi to 96 hpi. Due to the high number of differentially expressed genes at each time point, we confined our initial evaluation to the 10 genes showing the most significant up- or downregulation (fold change) in expression (Table 1).

**TABLE 1:** The genes that were most up- or downregulated at the intersection of the time points of the acute phase of LGTV infection^a^

Gene category	12 hpi versus:							
	24 hpi		48 hpi		72 hpi		96 hpi	
	Gene	Fold change	Gene	Fold change	Gene	Fold change	Gene	Fold change
Upregulated	*LGI1*	8.99	*NUPR1*	28.22	*NUPR1*	75.32	*NUPR1*	131.57
	*NUPR1*	7.93	*OSTN*	10.06	*KRTAP19-1*	16.31	*GAS5*	64.87
	*RPS17*	6.69	*LGI1*	8.73	*OSTN*	14.41	*IFIT1*	60.32
	*RPS29*	4.52	*CTH*	7.41	*ASS1*	13.82	*KRTAP19-1*	35.33
	*RPS3A*	4.51	*RPS17*	7.19	*CTH*	12.2	*RPL9*	25.02
	*RPL9*	4.48	*CHAC1*	7.04	*GAS5*	12.19	*CCNB1PIP1*	24.6
	*RSL24D1*	4.39	*GAS5*	7	*RPL9*	10.18	*RMRP*	21.9
	*RPL22L1*	4.37	*IFIT1*	6.83	*LGI1*	9.29	*LGI1*	20.69
	*RPL34*	4.26	*ASS1*	6.65	*RSL24D1*	9.26	*RPL26*	20.16
	*RPL36A*	4.23	*CCNB1PIP*	16.08	*CCNB1PIP*	18.74	*RPL39*	19.6
Downregulated	*CXCL1*	24.97	*CXCL1*	9.14	*CYP1B1*	16.64	*LGR5*	14.8
	*OAS3*	14.77	*OAS3*	7.95	*SHISA2*	13.81	*C22orf46*	14.46
	*NES*	6.07	*CYP1B1*	6.62	*NEAT1*	11.11	*DNAJC22*	12.97
	*FZD10*	4.81	*NEAT1*	6.22	*DOK3*	10.79	*CYP1B1*	12.94
	*MAGEA3*	4.64	*OSMR*	5.5	*FZD10*	10.63	*CRIPAK*	12.79
	*CYP1B1*	4.59	*NES*	4.83	*CRIPAK*	8.54	*DOK3*	12.73
	*NEAT1*	4.21	*SPEN*	4.74	*MAP1A*	8.27	*SHISA2*	12.48
	*SHISA2*	3.9	*FOXD4*	4.65	*C22orf46*	7.22	*TRIM66*	11.59
	*CRIPAK*	3.81	*LGR5*	4.64	*PCDH7*	7.17	*TRIM56*	11.48
	*HLA-DOA*	3.77	*SHISA2*	4.61	*TRIM66*	6.65	*MAP1A*	11.16

a A total of 519 genes were significantly differentially expressed at the intersection of all acute-phase time points, and these were analyzed with IPA to reveal those that were the most (fold change) up- or downregulated in expression at 24, 48, 72, and 96 hpi.

Among the genes that were downregulated in their expression, *CXCL1* (chemokine [C-X-C motif] ligand 1) (an IFN-stimulated proinflammatory gene) was the gene whose expression showed the greatest decrease at both 24 and 48 hpi, whereas *CYP1B1* (cytochrome P450, family 1, subfamily B, polypeptide 1) and *LGR5* (leucine-rich repeat containing G protein-coupled receptor 5) were the genes whose expression showed the greatest decrease at 72 and 96 hpi, respectively (Table 1).

Among the genes that were upregulated in expression, *LGI1* (leucine-rich, glioma inactivated 1) was the gene whose expression showed the greatest upregulation at 24 hpi, whereas *NUPR1* (nuclear protein, transcriptional regulator 1; associated with responses to metabolic stress) was the gene whose expression showed the greatest upregulation at 48, 72, and 96 hpi (Table 1).

### Pathway analysis of acute LGTV infection at specific time points.

**FIG 1:**
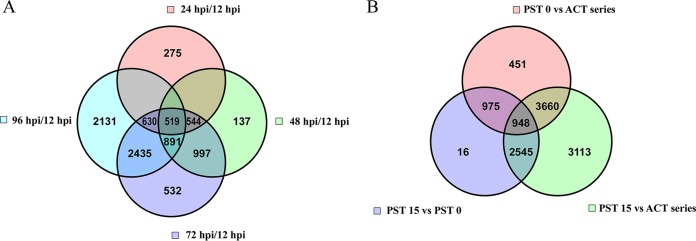
Analysis of common and unique gene expression differentials in acute and persistent LGTV infection. (A) Four-way Venn diagram of transcripts that were significantly differentially (>2-fold change and *q* of <0.05) expressed in acutely infected HEK 293T cells. The Venn diagram was constructed to identify the genes that were common to the acute phase and those that were different at each acute-phase time point. (B) Three-way Venn diagram comparing significantly differentially expressed genes in the whole acute phase (ACT series) and at initiation of persistence (PST-0) and late persistence (PST-15).

In order to evaluate gene expression throughout the acute infection, we constructed a Venn diagram to gain insight into the differential expression levels of genes that were unique or shared relative to the 12-hpi time point (Fig. 1A). This diagram revealed that 519 genes were common to all the time points. A total of 79% of the 519 genes were upregulated in expression, whereas the remaining 21% were downregulated. IPA of the 519 common genes revealed that the most statistically significant (*P* < 0.05) canonical pathways (ranked by *z* score) were the EIF2 signaling, regulation of eIF4 and p70s6k signaling, mTOR signaling, oxidative phosphorylation, and mitochondrial dysfunction pathways.

The numbers of genes unique at the 24, 48, 72, and 96 hpi time points were 275, 137, 532, and 2,131, respectively. At the 24-h time point, the most statistically significant (*P* < 0.05) canonical pathways associated with the 275 unique genes included G protein signaling mediated by Tubby, which is important in the function and maintenance of neuronal cells during development and differentiation. The genes involved in these pathways were mainly downregulated in expression.

At the 48-h time point, the 137 unique transcripts could be classified under the canonical pathways that included molecular mechanisms of cancer and glioblastoma multiforme signaling, pathways that are associated with dysregulation of cell survival and apoptosis. A higher proportion of the genes involved at this time point were downregulated in expression.

At 72 hpi, the 532 unique genes were also aligned with the canonical pathways associated with molecular mechanisms of cancer, cyclins, and cell cycle regulation, Wnt/β-catenin signaling, and retinoic acid receptor (RAR) activation. All these pathways are important in cell growth or transformation, and the majority of genes involved showed increased expression.

Finally, at 96 hpi, the canonical pathways associated with the 2,131 unique transcripts encompassed the protein ubiquitination pathway, RhoA signaling, CDP-diacylglycerol biosynthesis I, tRNA charging, and ephrin receptor signaling.

Taken together, these results suggest that acute LGTV infection affects the expression of genes involved in molecular and cellular functions, such as gene expression, cell death and survival and the cell cycle, and cellular assembly, organization, function, and maintenance, in a time-dependent manner.

### Transcriptome profile of HEK 293T cells persistently infected with Langat virus in comparison to acutely infected cells.

To determine the unique transcriptome profile of persistently infected 293T cells, we constructed another Venn diagram to delineate genes whose differential expression was unique to the persistent phase (Fig. 1B). At the point at which viral persistence was first established (PST-0), there were 451 genes expressed in manners different from those of the genes expressed in acutely infected cells or from those of the genes expressed in cells in late viral persistence (Fig. 1B). IPA revealed that the canonical pathways (ranked by *z* score) associated with those 451 genes included type II diabetes mellitus (DM) signaling, JAK/Stat signaling, Huntington’s disease signaling, the STAT3 pathway, and HIF1α signaling. For the canonical type II DM signaling, the genes with increased expression were *PIK3CB* (encoding the catalytic subunit of phosphatidylinositol 3-kinase [PI3K]), *TRADD* (TNFRSF1A-associated via death domain), *KCNJ11* (potassium channel inwardly rectifying subfamily J member 11), *SCL2AC* (solute carrier family 2/facilitated glucose transporter), *MAP3K14* (mitogen-activated protein kinase 14), and the oncogene *AKT2* (v-akt murine thymoma viral oncogene homolog 2), whereas the genes showing decreased expression were *MAPK10, PRKAB1* (protein kinase, AMP-activated, β1 noncatalytic subunit), *SOCS5* (suppressor of cytokine signaling 5), and *SOCS6*.

**FIG 2:**
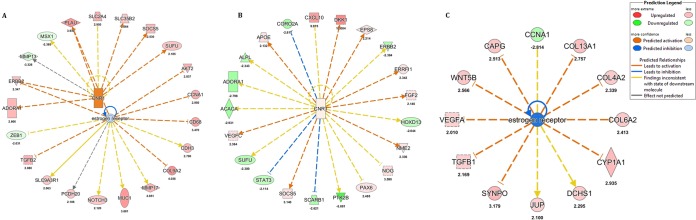
IPA depicting predicted activation of cannabinoid receptor (CNR1) and estrogen receptor (ESR) pathways in acutely and persistently infected cells at PST-0. (A) A combined CNR1 and ESR pathway showing that CNR1 was strongly activated and ESR was inhibited to a lesser extent. In these pathways, oncogenes *AKT2* and *ERRB2*, which favors cell survival, were upregulated. (B) In contrast, CNR1 was predicted to be activated to a lesser extent and the oncogene *ERBB2* was downregulated in acutely infected cells at 96 hpi. (C) The extent of inhibition of ESR was predicted to be higher in acutely infected cells at 96 hpi than in persistently infected cells at PST-0. Overall, the state of activation of CNR and ESR favored cell survival in persistently infected cells and favored cell death in acutely infected cells.

Results of upstream regulator analysis suggested that the increased expression of oncogenes *AKT2* and *ERBB2* or v-erb-b2 avian erythroblastic leukemia viral oncogene homolog 2 at the initiation of viral persistence (PST-0) was mediated via cannabinoid receptor 1 (CNR1) and the estrogen receptor (ESR) (Fig. 2A). CNR1 was predicted to be highly increased in expression, whereas expression of ESR was predicted to be slightly inhibited as measured by decreases in expression in cells at PST-0 in comparison to mock expression levels (Fig. 2A).

This result was in contrast to those seen with the acute phase of LGTV infection, in which it was predicted that CNR1 was less activated while ESR was more inhibited at 96 hpi relative to mock expression levels (Fig. 2B and C). Furthermore, expression of the *ERBB2* oncogene was downregulated in acute infection (Fig. 2B). In addition to the upregulation in expression of prosurvival oncogenes, we found that the expression of the proapoptotic gene *Bad* was downregulated in persistently infected cells but not in acutely infected cells (results not shown). Taken together, these findings suggested that the initiation of viral persistence was associated with the upregulation of expression of genes encoding prosurvival factors and downregulation of expression of genes encoding proapoptotic factors.

### Differential expression levels of innate immune cytokine-encoding genes in acutely and persistently infected HEK 293T cells.

Various elements of the innate immune response have been implicated in the cellular reaction to, and restriction of, flavivirus infection, including type I and type III interferon (IFN), IFN-stimulated tripartite motif (TRIM) proteins, mitochondrial activated antiviral signaling (MAVS), and interleukins (26, 27). It was of interest to survey our NGS results for transcriptomic information about these genes. Therefore, we examined differences in the expression levels of selected genes encoding cytokines involved in the innate antiviral immune response, specifically, IFN-β1, CCL5, CXCL10, and TNF-α, and further compared them in the acutely and persistently infected cells relative to mock-infected cells.

**FIG 3:**
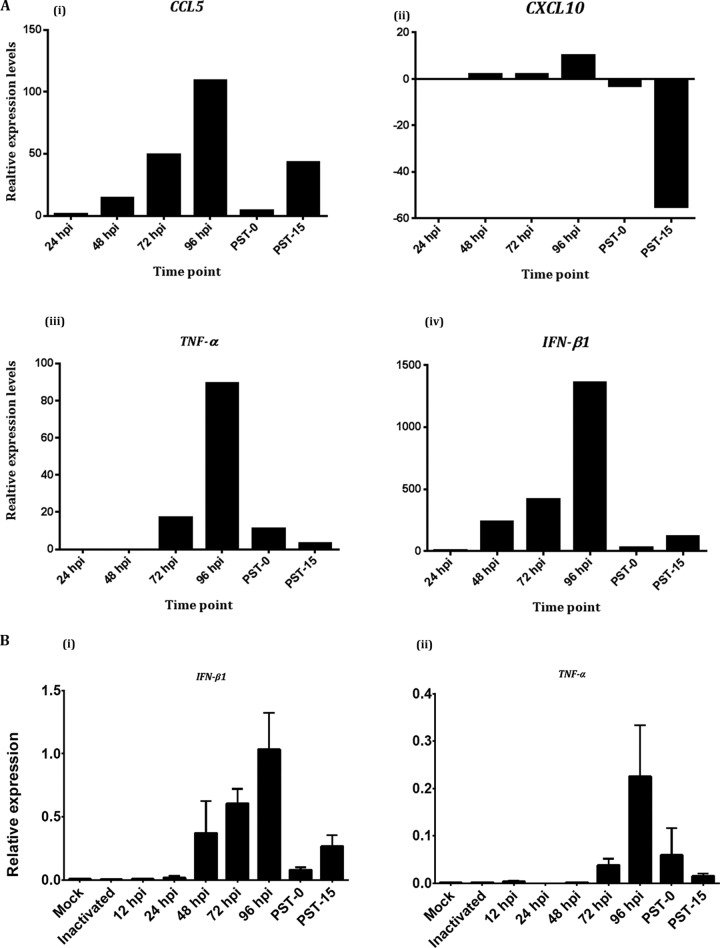
Analysis of the differential expression levels of selected genes. (A) Expression levels of innate immune cytokine-encoding genes in acutely and persistently infected HEK 293T cells. The bars were plotted from NGS data, and the expression levels were determined relative to those in mock-infected HEK 293T cells. (B) Validation of NGS transcriptome sequence data by qRT-PCR. mRNA transcripts of these genes were measured by both NGS and qRT-PCR, and the results correlated (Spearman’s) with *P* values of 4.0 × 10^−6^ for the *IFN-B1* gene and 1.8 × 10^−4^ for the *TNF-α* gene. The expression levels were relative to those of the *ADPGK* gene. The values represent the mean of results from 3 replicates, and the error bars represent standard deviations.

The perusal revealed that the expression levels of *IFN-β1*, *CCL5*, and *TNF* steadily increased as the acute phase progressed from 24 hpi, peaked at 96 hpi, and then sharply declined by the time of the initiation of viral persistence (PST-0) (Fig. 3A). The most dramatic changes were in the expression levels of *IFN-β1*, which increased from 7-fold at 24 hpi to 1,400-fold at 96 hpi (Fig. 3A, panel iv). However, at the initiation of viral persistence (PST-0), the expression levels of the IFN-β1 gene were increased only 29-fold relative to the expression levels at 12 hpi (Fig. 3A, panel iv). Expression levels of *CXCL10*, an IFN-inducible cytokine, followed a similar upward trend as the acute infection progressed from 24 to 96 hpi, but the expression levels were down 5- and 60-fold at PST-0 and PST-15, respectively (Fig. 3A, panel ii). Additional IPA suggested that the antiviral state associated with upregulation of *CXCL10* also involved *PARP1*, which is a marker of apoptosis when the encoded protein is cleaved (result not shown). Therefore, these results suggested that the innate immune response to LGTV was robust during acute infection but was decreased or relatively suppressed during viral persistence.

### Validation of deep-sequencing data.

To validate the expression findings from transcriptome sequencing, we selected the *IFN-β1* and *TNF-α* genes for quantitative reverse transcription-PCR (qRT-PCR) correlation. mRNA transcripts of these genes measured by both NGS and qRT-PCR showed statistically significant (Spearman’s) correlations, with *P* values of 4.0 × 10^−6^ and 1.8 × 10^−4^, respectively. The expression levels of *IFN-β1* and *TNF-α* were compared to those of the constitutively expressed reference ADPGK gene, and the data are shown in Fig. 3B. These results confirm that our NGS data appear to be accurate and valid and therefore could be used to infer biological relevance.

### Examination of apoptosis in HEK 293T cells acutely and persistently infected with Langat virus.

**FIG 4:**
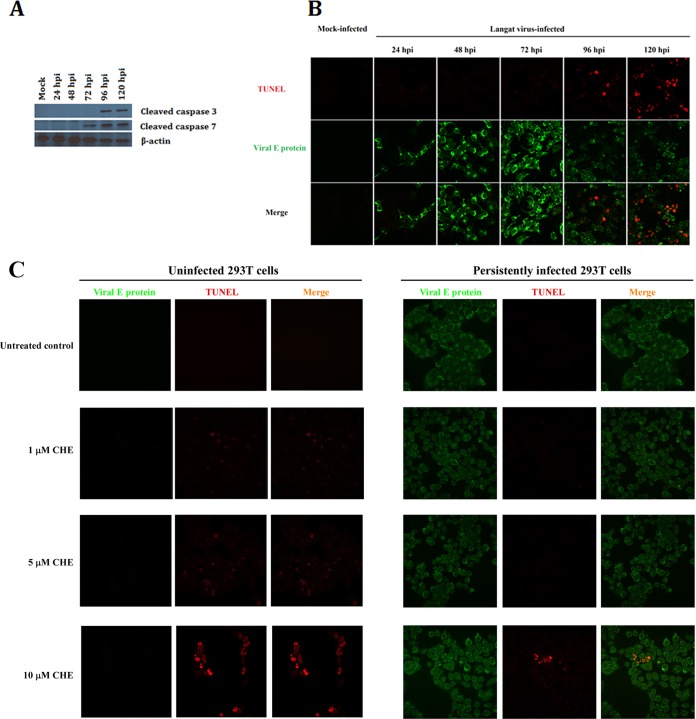
Characterization of the cell death mechanism of Langat virus-infected HEK 293T cells during acute infection. (A) Cleavage of effector caspases in acutely infected 293T cells. LGTV-infected cells were lysed in RIPA buffer and subjected to PAGE. Immunoblots were probed with mouse anti-caspase 3 and anti-caspase 7 antibodies followed by reaction with horseradish peroxidase (HRP)-labeled anti-mouse secondary antibody. Cleavage of caspases 3 and 7 was detected starting at 72 hpi. (B) Confocal microscopy images of TUNEL-stained ssDNA strand breaks (red stain) in Langat virus-infected (green stain) HEK 293T cells. ssDNA strand breaks were detected at 96 and 120 hpi (red nuclear staining). (C) Analysis of exogenous induction of apoptosis in uninfected and persistently infected 293T cells. Uninfected HEK 293T cells were sensitive to induction of apoptosis by all 3 concentrations of chelerythrine chloride (CHE) as shown by the presence of ssDNA breaks (red). Persistently infected cells were only minimally sensitive to induction of apoptosis by 10 µM CHE treatment.

The deep-sequencing data analysis suggested that apoptotic pathways were activated during acute infection but were downregulated in expression in persistent infection. In an effort to correlate the NGS findings with virus biology, we infected monolayers of HEK 293T cells with LGTV and inspected infected cells daily by light microscopy. The cultures exhibited normal morphology until 72 hpi, when signs of stress, i.e., rounding up and increased refraction, were observed along with additional macroscopic signs of stress observed at 96 hpi. By 120 hpi, the majority of cells had undergone a frank lytic crisis, detached from the culture vessel, and died. The detection of cleaved caspases 3 and 7 implicated apoptosis in this lytic phase (Fig. 4A). The development of apoptosis was further supported by the demonstration of nuclear ssDNA breaks using a terminal deoxynucleotidyltransferase-mediated dUTP-biotin nick end labeling (TUNEL) assay (Fig. 4B). Consequently, the results confirmed that apoptosis was the proximal cause of the lytic crisis associated with acute LGTV infection. Notably, the cells in the small number that survived were positive for viral E protein, indicating that some of the acutely infected cells evaded apoptosis.

These data suggested that the persistently infected cultures derived from acutely infected cells that had evaded apoptotic death. This further suggested that the persistently infected cells had become resistant to apoptosis. To test this hypothesis, we exposed persistently infected cells and uninfected cells to chelerythrine chloride (CHE), an exogenous inducer of apoptosis that operates via protein kinase C (PKC), the mitochondrial pathway, and the Bcl2 family of proteins. We readily detected ssDNA breaks in the nuclei of uninfected HEK 293T cells after treatment with 1, 5, or 10 µM CHE, thus indicating that the chemical was effective at inducing apoptosis in these cells (Fig. 4C). In contrast, no ssDNA breaks were detected by TUNEL assay in persistently infected cells after treatment with 1 or 5 µM CHE (Fig. 4C), an observation consistent with resistance to induction of apoptosis. However, following treatment with 10 µM CHE, a small percentage of persistently infected cells tested positive for ssDNA breaks (Fig. 4C), suggesting that resistance of these cells to induction of apoptosis was not absolute but was dose dependent. Thus, it was likely that persistently infected cells had acquired resistance to apoptosis involving, at least, the mitochondrial pathway. These findings corroborated our results from transcriptome profiling which suggested that apoptosis was blocked in persistently infected cells.

## DISCUSSION

The biology of vector-borne flaviviruses (VBFVs) is exceedingly complex. These agents of severe, sometimes fatal disease have evolved mechanisms to replicate and persist in both mammalian and arthropod hosts. The basic biological features of tick-borne flavivirus (TBFV) persistence in the arthropod host are well described, but details about persistence in mammalian systems *in vitro* have just recently been reported, and the precise molecular and genetic mechanisms remain to be elucidated (16, 28–34). Acute TBFV infection of HEK 293T cells is accompanied by a dramatic lytic crisis, but a small fraction of the surviving cells repopulate and become persistently infected; cultures of persistently infected cells can be maintained for extended lengths of time (24). Maintenance of the persistent infection is associated with the presence of defective interfering virus particles (DIPs), but the DIPs are not a feature of the acute phase and are not present at the initiation of persistence. In the current study, we directed our attention to changes in the cellular transcriptome during acute infection as well as during the initiation and maintenance of persistent infection. We used next-generation sequencing of mRNA at selected time points, analyzed the data for significant differential expression levels, and performed pathway analysis of the potential functional categories affected. Our results indicated that there were stark differences in the levels of expression of genes between the acute and persistent phases of infection and that a number of genes were uniquely differentially expressed at the initiation of persistence.

Elimination of virus-infected cells by apoptosis is often effected by the innate immune response, which is considered a vanguard against RNA virus infection. A hallmark of the host response to acute viral infection is the elaboration of IFNs (35–39). We observed increases in expression levels of innate immune cytokine-encoding genes during the time course of the acute phase (Fig. 3); this finding suggested that the innate immune response leading to apoptosis in infected HEK 293T cells was intact and functional throughout acute infection. However, *IFN-β1* expression seemed to increase slightly at PST-15, suggesting that there may be continued, but controlled, provocation of the pathogen recognition receptors by LGTV-associated molecular patterns.

We also identified some gene expression changes associated with cellular metabolic responses in acute LGTV infection. Specifically, *NUPR1* gene expression was the most upregulated at the intersections of all acute infection time points (Table 1). This suggested that the transcription of genes encoding metabolic functions was negatively affected (40, 41). Increased levels of NUPR1 are required for the expression of DNA damage- and cell cycle-related genes under low-glucose and hypoxic conditions (40). NUPR1 regulates p21 and interacts with TP53, which was also among the transcription regulators at 48 and 72 hpi. NUPR1 overexpression appears to be protective against metabolic-stress-mediated apoptosis (40, 42). However, TP53 mainly functions as a tumor suppressor by arresting cell growth and inducing apoptosis (43, 44), and we speculate that it could have superseded the effect of NUPR1 on apoptosis at the end of the acute infection. Upregulation of the expression of the NUPR1 gene was also observed in mouse brains infected with JEV or WNV (45), suggesting that TBFVs and encephalitogenic MBFVs share this ability to induce metabolic stress during infection. Endoplasmic reticulum (ER) stress and an unfolded protein response also add to the biochemical burden of infection by VBFV due to the fact that these viruses use the ER as the primary site for protein synthesis, RNA replication, and virion maturation (46, 47).

At the crucial time point at which persistence was initiated in our studies (PST-0), 451 genes were significantly uniquely differentially expressed (Fig. 2B). Ingenuity Pathway Analysis (IPA) implicated type II diabetes and Huntington’s disease signaling as canonical pathways at that time point. Although the involvement of these pathways might appear curious, close inspection of specific genes indicated that expression levels of prosurvival genes, such as *AKT2* and *ERBB2*, were upregulated (Fig. 2A). AKT2 is one of three closely related serine/threonine kinase isoforms (AKT1, AKT2, and AKT3) and is closely linked to type II diabetes, and it is also overexpressed in cancers at a higher frequency than AKT1 (48, 49). Ablation of AKT2 has been reported to cause autophagy in MDA-MB231 cells through the downregulation of p70s6k and dysregulation of mitochondria, where AKT2 is localized (50). Our analyses predicted that p70s6k signaling and mitochondrial dysfunction were common features of HEK 293T cells acutely infected with LGTV but also that these pathways were inhibited in persistently infected cells. These observations suggested that AKT2’s interactions with these pathways could be important factors contributing to the death of acutely infected cells or to evasion thereof by persistently infected cells.

Gene products of other viruses, such as polyomavirus, hepatitis B virus, human papillomavirus, and Epstein-Barr virus, activate the PI3K/AKT pathway through various mediators to suppress apoptosis in infected cells (51–54). Short-term activation of the PI3K/AKT pathway leads to inhibition of apoptosis in cells infected with JEV, DENV, encephalomyocarditis virus, and respiratory syncytial virus (55–57). Although TBFVs are known to be highly cytopathic, persistently infected HEK 293T cells seem to have attained an apoptosis resistance phenotype. Clearly, further mechanistic studies to elucidate the precise role of the oncogenes *AKT2* and *ERBB2* and their interaction with viral proteins in TBFV persistence are warranted.

Our results clearly demonstrate that suppression and evasion of the innate immune system-induced antiviral state in a subset of cells were crucial for the initiation and maintenance of TBFV persistence. Therefore, it seemed logical that proapoptotic genes, such as *Bad* and *IFN-β1* (Fig. 4), were downregulated in persistently infected cells. Activation of these factors could lead to antiproliferation and apoptosis (58), thus abolishing a viral persistence state.

In summary, we extensively characterized the transcriptome of HEK 293T cells during acute and persistent infection with a tick-borne flavivirus. Acutely infected cells undergo metabolic stress, as suggested by the *NUPR1* gene being the most highly expressed gene in this acute phase. Most important was the discovery of a unique transcriptome signature composed of 451 transcripts that were differentially expressed only in persistently infected cells. This profile was characterized by the upregulation of the expression of prosurvival or oncogenic genes *AKT2* and *ERBB2* and by the downregulation of the expression of proapoptotic genes such as *Bad* and *IFN-β1*. These results provide a molecular basis for conducting additional research aimed at unraveling the mechanism of tick-borne flavivirus persistence and the specific viral proteins that appear to initiate this phenotype.

## MATERIALS AND METHODS

### Cells and virus.

Human embryonic kidney (HEK) 293T cells (ATCC) were maintained in complete Dulbecco’s modified Eagle’s medium (DMEM) containing 10% fetal bovine serum (Gibco, Life Technologies) and 50 µg/ml gentamicin (Gibco, Life Technologies). Langat TP21 virus (LGTV) was rescued by transfection of African green monkey kidney cells (Vero; ATCC) with viral RNA obtained from *in vitro* transcription of a cDNA clone as described previously (24). For some studies, LGTV was heat inactivated by incubation of virus at 56°C for 45 min. Inactivation was validated with an immunofocus assay using an anti-E (11H12) antibody (a kind gift from Connie Schmaljohn, USAMRIID, Fort Detrick, MD).

### Infection of HEK 293T cells.

To characterize the host cell transcriptome profiles of the acute phase of LGTV TP21 infection, 1.5 × 10^6^ HEK 293T cells in 25-cm^2^ flasks (Nunc) were infected in triplicate with Langat TP21 virus at a multiplicity of infection (MOI) of 5. For 12 h samples, 1.5 × 10^6^ 293T cells were infected at a MOI of 10. Some cells were separately exposed to heat-inactivated LGTV TP21 virus at a MOI of 10 and harvested after 12 h. Triplicate control cells were mock infected with virus-free DMEM. All infected and mock-infected cells were incubated in complete DMEM at 37°C in 5% CO_2_ and harvested for total RNA extraction at 12, 24, 48, 72, and 96 h postinfection (hpi). Persistently infected cells were generated as described previously (24).

### RNA extraction and host cell transcriptome sequencing.

At the various acute-phase time points mentioned above, cells were washed twice with cold phosphate-buffered saline (PBS; Gibco, Life Technologies) and lysed with 1 ml of TRIzol reagent (Invitrogen). Total RNAs were extracted and analyzed for both quality and quantity following protocols previously described (59). Host mRNA sequencing used Illumina TruSeq RNA Sample Preparation kit v2 (Illumina, San Diego, CA), beginning at the poly(A) selection step. Libraries were bar coded, the number of amplification cycles was reduced to 10, and libraries were run as a pool across all 8 lanes of a HiSeq 2500 sequencer (Illumina, San Diego, CA).

### Transcriptome data analysis.

The resulting sequence reads were trimmed of adaptor and poor-quality sequences and mapped to build hg19 of the human genome using Top hat 2 software (60). The sequence data were submitted to the National Center for Biotechnology Information Small Read Archive (http://www.ncbi.nlm.nih.gov/Traces/sra/; study no. SRP067516). Transcript quantification and differential gene expression analysis were performed using the Cufflinks software suite (61). The data were filtered at >2-fold change (up or down) and a false-discovery-rate (FDR)-corrected *P* value (*q* value) of <0.05. Filtered data were analyzed to generate pathways, networks, heat maps, and functional inference using Qiagen’s Ingenuity pathway analysis (IPA) software (Qiagen, Redwood City, CA). Additional biological process classifications were performed using protein analysis through evolutionary relationships (PANTHER), which assigns function by inferring large networks of proteins that interact to accomplish a process at the cellular or organism level (http://pantherdb.org/) (62–64).

### Validation of transcriptome sequencing data.

All RNA samples at each acute-phase time point studied by NGS were included in the quantitative PCR (qPCR) validation analysis. The constitutive ADPGK gene was selected for this validation on the basis of low coefficients of variation (CV) across treatments, gene functions, and expression levels. The validation genes selected were the IFN-β1 and TNF-α genes. RNAs used for this validation showed DNA contamination of less than 0.01% of the RNA used as the template. cDNA was synthesized with a SuperScript Vilo cDNA synthesis kit, according to the protocol of the manufacturer (Invitrogen, Carlsbad, CA). cDNAs were purified according to a QIAquick 96-well protocol (Qiagen, Valencia, CA), with a modified centrifugation protocol (65).

Aliquots of target cDNA were pooled and used as the template to run a qPCR amplification efficiency test for both reference gene primer and probe sets in combination with each validation primer and probe set. Primers and probe sets were purchased from Biosearch Technologies (Petaluma, CA). Two sets of primers and probes for the IFN-β1 gene were used due to difficulty in design of this gene sequence (Table 2). Invitrogen’s Express qPCR Supermix with premixed ROX (Life Technologies, Carlsbad, CA) was used to perform the assays. The multiplex reactions were carried out in 20-µl reaction mixtures, and qPCR amplifications were carried out at 50°C for 2 min, at 95°C for 2 min, and for 55 cycles of 95°C for 15 s and 60°C for 1 min. Data were analyzed using 7900HT version 2.4 sequence detection system software according to the recommendations of the manufacturer (Life Technologies, Carlsbad, CA).

**TABLE 2:** Primers and probes used for qRT-PCR validation of NGS data^a^

Transcription gene	Exon(s)	Sequence	5′ modification	3′ modification
TNF	2, 3, and 4	CCAGGCAGTCAGATCATCTTCTCTCTCAGCTCCACGCCATTGCCCGAGTGACAAGCCTGTAGCCCAT	6-FAM	BHQ-1
IFN-B1	1	CTTTCCATGAGCTACAACTTGCTTATCTCCTCAGGGATGTCAAAGTTCCAATTTTCAGTGTCAGAAGCTCCTGTGGC	6-FAM	BHQ-1
IFN-B1	1	CAGCAATTTTCAGTGTCAGAAGCTCATCCTGTCCTTGAGGCAGTATTAAGCCTCCCATTCAATTGCCA	6-FAM	BHQ-1
ADPGK	4 and 5	CTTCTTGATGACAATGTCTTTGTTCTTTAACTGGCCCCACTCCTCATCCACTTCCTGCAATGACTCTGGT	CFG540	BHQ-1

a 6-FAM, 6-carboxyfluorescein; BHQ-1, black hole quencher 1.

### Evaluation of apoptosis.

Apoptosis was evaluated using TUNEL staining (66). LGTV-infected HEK 293T cells at various acute-phase time points were washed twice with PBS and fixed with 4% paraformaldehyde for 1 h at room temperature. Fixed cells were washed with PBS and permeabilized with 0.1% Triton X-100–0.1% sodium citrate at room temperature for 20 min. To visualize the viral protein, permeabilized cells were treated with a TMR-Red TUNEL reaction mixture (Roche) and incubated with an anti-E (11H12) antibody diluted to 1:1,000 at 37°C in a humidified chamber in the dark for 1 h. The treated cells were washed three times with PBS and incubated with an Alexa Fluor 488-labeled secondary antibody (diluted to 1:1,000) at 37°C for 1 h. The cells were washed 4 times with PBS and mounted using Prolong gold Antifade reagent (Life Technologies). Visualization was performed at 488 nm (green) and 561 nm (red) with a Zeiss LSM710 confocal microscope.

Apoptosis was also evaluated by examining caspase cleavage. HEK 293T cells (4 × 10^6^) were infected with a Langat TP21 virus at a MOI of 5 for 1 h at 37°C. Infected cells were incubated at 37°C and 5% CO_2_ followed by harvesting at 24, 48, 72, 96, and 120 hpi. Cells were subjected to trypsinization and washed with cold PBS with centrifugation at 5,000 rpm for 3 min at 4°C. The pellet was lysed with radioimmunoprecipitation assay (RIPA) buffer (Abcam) for 30 min on ice. A bicinchoninic acid (BCA) assay was performed, and equivalent protein quantities were prepared for polyacrylamide gel electrophoresis. Membranes were blocked with 5% skimmed milk in 1X Tris buffered saline (TBS). The blots were probed with mouse-generated antibodies against cleaved caspase 3 and caspase 7 (Cell Signal), and a mouse anti-β actin antibody (Sigma) was used for controls.

To exogenously induce apoptosis, uninfected and persistently infected HEK 293T cells were exposed to 1, 5, or 10 µM chelerythrine chloride (Santa Cruz Biotechnology) dissolved in DMEM for 30 min at 37°C. Following this treatment, cells were washed with PBS, fixed, stained for ssDNA breaks with TUNEL and viral E protein, and examined by confocal microscopy as described above.
